# Adsorption of phenol using adsorbent derived from *Saccharum officinarum* biomass: optimization, isotherms, kinetics, and thermodynamic study

**DOI:** 10.1038/s41598-023-42461-y

**Published:** 2023-10-26

**Authors:** Upendra R. Darla, Dilip H. Lataye, Anuj Kumar, Bidhan Pandit, Mohd Ubaidullah

**Affiliations:** 1 Department of Civil Engineering, Visvesvaraya National Institute of Technology, Nagpur, 440010 India; 2 Department of Chemistry, GLA University, Mathura, 281406 India; 3 Department of Materials Science and Engineering and Chemical Engineering, Universidad Carlos III de Madrid, Avenida de la Universidad 30, 28911 Leganés, Madrid Spain; 4 Department of Chemistry, College of Science, King Saud University, P.O. Box 2455, 11451 Riyadh, Saudi Arabia

**Keywords:** Environmental sciences, Chemistry

## Abstract

The present research shows the application of Taguchi's design of experiment approach to optimize the process parameters for the removal of phenol onto surface of *Saccharum officinarum* biomass activated carbon (SBAC) from an aqueous solution to maximize adsorption capacity of SBAC. The effect of adsorption parameters viz. adsorbent dose (m), temperature (T), initial concentration (C_0_) and mixing time (t) on response characteristics i.e., adsorption capacity (q_t_) has been studied at three levels by using L_9_ orthogonal array (OA) which further analyzed by variance analysis (ANOVA) for adsorption data and signal/noise (S/N) ratio data by using ‘larger the better’ characteristics. Using ANOVA, the optimum parameters are found to be m = 2 g/L, C_0_ = 150 mg/L, T = 313 K and t = 90 min, resulting in a maximum adsorption capacity of 64.59 mg/g. Adopting ANOVA, the percentage contribution of each process parameter in descending order of sequence is adsorbent dose 59.97% > initial phenol concentration 31.70% > contact time 4.28% > temperature 4.04%. The phenol adsorption onto SBAC was best fitted with the pseudo-second-order kinetic model and follows the Radke-Prausnitz isotherm model. Thermodynamic parameters suggested a spontaneous, exothermic nature and the adsorption process approaches physisorption followed by chemisorption. Hence the application of Taguchi orthogonal array design is a cost-effective and time-efficient approach for carrying out experiments and optimizing procedures for adsorption of phenol and improve the adsorption capacity of SBAC.

## Introduction

Water pollution is a significant concern in India and many other parts of the world. Rapid industrialization, population growth, urbanization and inadequate wastewater treatment infrastructure contribute to the country’s water pollution problem^1^. The readily accessible natural sources of drinking water are at danger from organic pollutants like phenol and its derivatives. Phenol is chemical compound that is commonly used in industrial processes, including the production of plastics, resins, and pharmaceuticals^2^. It can enter water bodies through industrial discharge, improper disposal of chemicals, or accidental spills. Presently, phenol is produced at a global rate of around 6 million tonne per year, with a rapidly growing tendency^3^. Phenolic compounds in wastewater pose a severe discharge problem due to their high toxicity and low biodegradability^4^. Since the solubility of phenol is high in water, excessive inhalation or exposure may end in coma, seizure, nausea, cyanosis, diarrhea and other adverse effects^5^. According US environmental protection agency guidelines, the permissible limit for phenol is 0.1 mg/L in wastewater and 1 µg/mL in water supplies respectively^6^. Permissible limit for phenol in drinking water as per Bureau of Indian Standards is 0.002 mg/L^7^. Therefore, it is crucial to treat water and wastewater to remove phenol and its derivatives before discharging them into water bodies.

Numerous technologies have indeed been developed for the removal of phenols from aqueous solutions, such as adsorption^8^, biological treatment^9^, photocatalytic degradation^10^, chemical oxidation^11^, uptake by ion exchange resins^12^, precipitation^13^, electrochemical incineration^14^ and membrane separation^15^. Activated carbon (AC) has indeed been widely recognized as one of the most effective methods for the removal of phenols from aqueous solutions. Its use in adsorption processes offers several advantages, including simplicity, availability, and high efficiency^16^. Many adsorbents such as commercial activated carbon^3^, carbon nanotubes^17^, guava tree bark^18^, graphene oxide^19^, rice husk^20^, peanut shells^21^, clay^22^, banana peel^4^, babul sawdust^23^ and orange peel ash^24^ are used.

India is one of the world's major sugarcane (saccharum officinarum) growers, output reaching a record of 376.9 million tonnes in 2017–2018. This placed India as the second-highest producer of sugarcane globally, after Brazil^25^. A significant volume of sugarcane bagasse (SB) is produced and often it is incinerated on agricultural sites or disposed of in landfills. SB is the biodegradable organic substance, and it can be transformed into a potential adsorbent due to the presence of carbonyl and hydroxyl groups, which can absorb the phenol from aqueous solution. Sugarcane bagasse is chemically modified with zinc chloride solution named as SBAC and used as adsorbent for the phenol adsorption^26^.

Taguchi's optimization technique, also known as Taguchi's robust design, has been widely adopted by researchers and scientists in various fields, particularly in manufacturing and processing, to produce high-quality products and improve process efficiency^27^. While it has been successfully applied in areas such as polymer optimization, biotech processes, animals and birds' social behavior, and even electro degradation of herbicides^28^. There has been limited contribution from researchers in utilizing the Taguchi method for optimizing sorption process parameters in the field of adsorption^29,30^.

The current study is adopting Taguchi's optimization technique, which can be effectively optimizing parameters such as adsorbent dosage, mixing time, temperature, pH, agitation speed, and initial phenol concentration. This approach helps to maximize the phenol adsorption capacity of SBAC while minimizing the number of experimental runs and associated costs. To optimize the process parameters and achieve maximum phenol uptake, Taguchi's optimization technique with an L_9_ (3^4^) orthogonal array was employed. The study also includes investigations into the isotherm equilibrium, kinetics, and thermodynamics of the adsorption process to gain a better understanding of phenol interaction with the SBAC. Further SEM, FTIR, BET, and proximate analyses are conducted to examine the surface morphology of the adsorbent. This study provides valuable insights into the potential of utilizing sugarcane bagasse as an environmentally friendly solution for water treatment.

## Material and methods

### Chemicals and reagents

Analytical reagent grade chemicals are used without additional purification and the specifics of the substances used in the study can be found in Table S1. To prepare the reagents, double-distilled water (DDW) was used. To prepare phenol stock solution, 1 g of anhydrous phenol is dissolved in 1 L of DDW, resulting in 1000 mg/L concentration. To obtain the concentrations of 50, 100, and 150 mg/L, the stock solution is further diluted using DDW. It is mentioned that the chemicals were procured from the Central Scientific Company in Nagpur, India.

### Preparation and characterization of SBAC

Saccharum officinarum (sugarcane) is one of the major commercial crops in India and it is planted thrice a year in October, March and July depending on the part of the country without the addition of fertilizers and under a controlled watering regime^31^. Sugar cane bagasse is a lignocellulosic fibre residue obtained from sugar cane culm after the culm is milled and the juice is extracted. The average composition of sugar cane is 65–75% water, 11–18% sugars, 8–14% fibres and 12–23% soluble solids. The cane basically consists of juice and fiber^32^. The sugar cane bagasse has the following composition (by weight): cellulose, 41.8%; hemicellulose (as pentosane), 28.0%; lignin, 21.8%^33^. In the present study, agricultural and agro-industrial waste materials like sugarcane bagasse were obtained from Mahatma Phule local market of Nagpur district, Maharashtra, India. The material was undergone thorough cleaning with tap water to eliminate the soil/residue, and afterwards it was subjected to sun drying for about 48 h. The dried sugarcane bagasse was grounded into fine particles and sieved for acquiring particles in the range of 850–300 µm. Sieved material was blended with 0.1 M zinc chloride solution (ZnCl_2_) in 1:0.25 (g:mL) proportion. This mixture was placed in muffle furnace for carbonization at a temperature of 673 K for 60 min. The obtained charred sample was washed many times by doubled distilled water (DDW) to make it free from the acid and dried at 378 K for about 3 h. The dried carbon passing through 600 µm sieve and retaining on 150 µm sieve (ASTM 11–70) was utilized for adsorption study”^26^. The proximate analysis of adsorbent was performed according to the BIS 1350-1 (1984) standard. “Scanning electron microscopy (SEM) was carried out using a JSM-7610F instrument from Japan to examine the surface morphologies of SBAC. Fourier transform infrared spectrometry (FTIR) was employed using an IR Affinity-1 instrument (Miracle 10, Shimadzu, Japan). The FTIR analysis covered the spectral range of 400–4000 cm^−1^ to study the surface functional groups present on SBAC. The Brunauer–Emmet–Teller (BET) method is used to measure the surface area and pore characteristics of SBAC. Nitrogen adsorption at 77 K was performed using a Quantachrome Nova touch 1.1analyzer.” Approximately 0.35 g of dry powder sample was degassed for about 2 h at 200 °C before conducting the BET analysis. Origin Pro (2022b) software was used for the processing of the raw data obtained from the characterization.

### Taguchi’s method

The Taguchi’s L_9,_ orthogonal array (OA) is used to optimize the adsorption parameters affecting the adsorption capacity (q_t_) of SBAC. The controllable process parameters considered are adsorbent dose, initial phenol concentration, temperature, and mixing time, denoted as factors A, B, C, and D, respectively. Each of these parameters is tested at three different levels, labeled as L1, L2, and L3 shown in Table S2. The study did not consider two other parameters, initial pH and agitating speed, as they were previously found to have minimal influence on the phenol adsorption onto SBAC^26^. To determine the number of experiments needed for optimization, the authors applied the partial factorial method. According to this method, the number of experiments, denoted as E, can be calculated using the formula E = 2 k + 1, where k represents the total factors and their interactions. In this case, since there were four parameters, the number of experiments is determined as E = 9. Thus, an L_9_ orthogonal array was chosen, which is designed for three-level testing conditions and can be seen in Table S3. Based on the selected parameters and their levels in Table S2, a Design of Experiment (DOE) is created, specifying the values for each factor level. These factor level values are provided in Table S3.

### Adsorption experiments

According to DOE, there are nine experimental runs and each run is repeated thrice to eradicate experimental and analytical errors. Experiments are carried in a 250 ml conical flask at fixed volume of phenol (50 mL) while altering m and C_0_ in accordance with DOE. All the studies are conducted by keeping the samples in an orbital shaking incubator [REMI, model: CIS 24-BL] with a set agitation speed 150 rpm at temperature T for time t. the effluent absorbance of phenol measured by using UV–VIS spectrophotometer [Shimadzu, model: UV-2450] at 269.5 nm (the maximum wavelength of absorption of phenol is 270 nm^23^). To determine the quantity of phenol removal and adsorption capacity SBAC, the following equations are used^3^:

1 $$\% \;{\text{Removal}} = \frac{{(C_{0} - C_{e} )}}{{C_{0} }} \times 100$$

2 $$q_{t} = \frac{{(C_{0} - C_{e} )\;{\text{V}}}}{m}$$

where, V phenol volume (L), C_0_ and C_e_ are the influent and effluent concentration of phenol (mg/L), q_t_ phenol uptake on SBAC (mg/g), and m adsorbent dosage (g).

### Experimental data analysis

ANOVA (Analysis of Variance) and S/N (Signal-to-Noise) ratio are used to analyse the data collected from the experimental study. The S/N (signal-to-noise) ratio is used to assess the impact of uncontrollable factors, also known as noise factors^34^. states that the S/N ratio helps reduce the variability caused by these noise factors. The S/N ratio is a measure of the quality of a function or process. The larger the ratio, the better the function performs relative to the noise or errors present. Three types of S/N ratio which are^34^:

Smaller the better

3 $$\frac{S}{N} = - 10\log \left[ {\frac{1}{n}\sum\limits_{i = 1}^{n} {y_{i}^{2} } } \right]$$

Larger the better

4 $$\frac{S}{N} = - 10\log \left[ {\frac{1}{n}\sum\limits_{i = 1}^{n} {\frac{1}{{y_{i}^{2} }}} } \right]$$

Nominal the better

5 $$\frac{S}{N} = 10\log \frac{{\mu^{2} }}{{\sigma^{2} }}$$

Equation (3) is used to find the larger the better S/N ratio in experimental design and analysis. It quantifies the relationship between the mean (μ) and variance (σ^2^) of the observed response variables (y_i_) for a given number of trials (n). After calculating the S/N ratios, the data are often further analyzed using analysis of variance (ANOVA) techniques. When applying ANOVA to experimental data, an orthogonal array (OA) can be used to allocate factors to experimental trials efficiently. Each column of the OA represents a specific factor and its associated degrees of freedom (DOF), which is equal to the number of levels minus one. The array must meet the criterion that the total DOF of OA is more than or equal to the total DOF of the experiment. The methodology for analyzing experimental data is described in detail^35,36^. ANOVAs analysis conducted on raw data to find out the optimum conditions and the mean of response (*μ*), for example A, B, C, and D are the parameters optimized at one, three, two and one level respectively then *µ* calculates using the formula:

6 $$\mu = \frac{T}{N} + \left( {A_{2avg} - \frac{T}{N}} \right) + \left( {{\text{B}}_{3avg} - \frac{T}{N}} \right) + \left( {{\text{C}}_{2avg} - \frac{T}{N}} \right) + \left( {{\text{D}}_{1avg} - \frac{T}{N}} \right)$$

whereas T is the grand total of all results, N is the total number of results; A_1avg_, B_3avg_, C_2avg_ and D_1avg_ are the average response values.

Based on the average outcomes of the studies, *µ* is calculated. The optimized result was examined using a confidence interval (CI), which shows where a statistical parameter's value falls in relation to where it should fall given a certain degree of confidence. The CI is classified into two categories: CI_CE_, which is only applicable to a sample group or set of experiments under certain circumstances, and CI_POP_, which is the confidence interval for the whole population^30^.

7 $$CI_{POP} = \sqrt {\frac{{F_{\alpha } (1,f_{e} )\;{\text{V}}_{e} }}{{n_{eff} }}}$$

8 $$CI_{CE} = \sqrt {F_{\alpha } (1,f_{e} )V_{e} \left[ {\frac{1}{{n_{eff} }} + \frac{1}{R}} \right]}$$

“where F_α_ (1, *f*_e_) = the F-ratio at a confidence level of (1 − α) against DOF 1 and error of DOF (*f*_*e*_) its value can find in F table, V_e_ = error variance (from pooled ANOVA)”

9 $$n_{eff} = \frac{N}{1 + [Total \;DOF\; associated \;in \;the\; estimated\; of\; the \;mean]}$$

where R is the sample size for confirmation experiment, as R approaches infinity, meaning the sample size approaches the entire population (N), the term 1/R approaches zero. This implies that the influence of the sample size on the calculation of the confidence interval diminishes, and the CI_CE_ converges to the Confidence Interval for the Population (CI_POP_). On the other hand, as R approaches 1, indicating a very small sample size relative to the population, the term 1/R becomes large. Consequently, the CICE becomes wider, indicating a larger margin of error and greater uncertainty in the estimate of the population proportion^37,38^. The confirmation test will be performed at optimal conditions for parameters, with the average of the results confirmed at 95% confidence intervals. If Taguchi's model value and confirmation experiment value are within the 95% confidence interval, this confirms the validity of the optimum parametric values obtained from Taguchi's design.

## Results and discussion

### Adsorbent characterization

**Figure 1:**
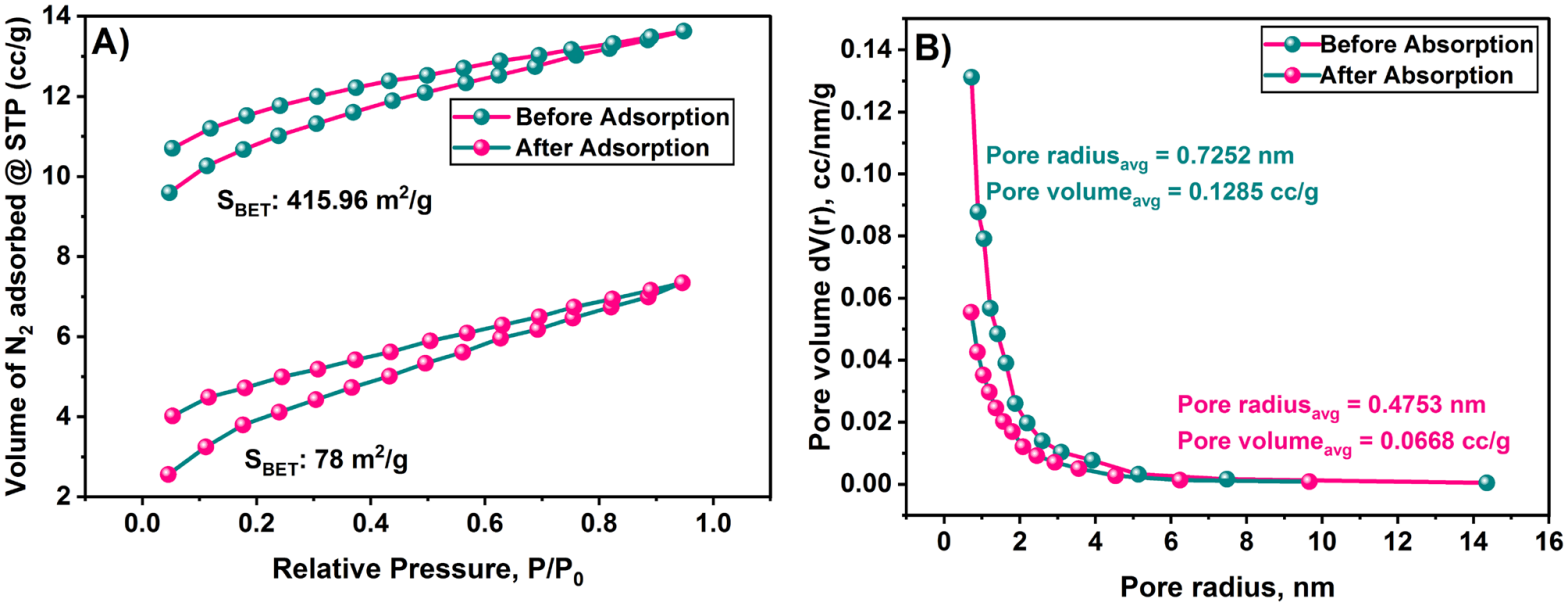
(**A**) N_2_ adsorption–desorption isotherms for before and after adsorption and (**B**) pore volume versus pore size distribution graph of SBAC before and after adsorption.

The N_2_ adsorption–desorption tests using the BET-BJH method indicate a type-II isotherm, which suggests a multilayer adsorption phenomenon. This information is derived from Fig. 1.

Before adsorption, the surface area of the SBAC is measured to be 415.96 m^2^/g. However, after adsorption, the surface area decreases significantly to 78.33 m^2^/g. The reduction in surface area is approximately 337.63 m^2^/g or 81.16% of the initial value. The efficient adsorption of phenol is attributed to the observed surface area and microporous structure of the SBAC. This indicates that the SBAC material is capable of effectively capturing and removing phenol through adsorption process.

The proximate analysis of adsorbent material was conducted according to the BIS 1350-I (1984) standard. The results of this analysis are shown in a Table S4. The analysis provides important information about the composition of the adsorbent, which can be useful in determining how to manage the material after it has been used for the adsorption process. Lower moisture content and volatile substance are desirable because it can increase the calorific value of the material. The amount of ash content can provide insights into the management of the ash after the adsorbent has been used. The high content of fixed carbon in the adsorbent indicates a better calorific value, making it suitable for incineration or disposal methods that involve burning. However, it's important to consider the emissions of potentially toxic substances during incineration to ensure proper environmental management^4^.

**Figure 2:**
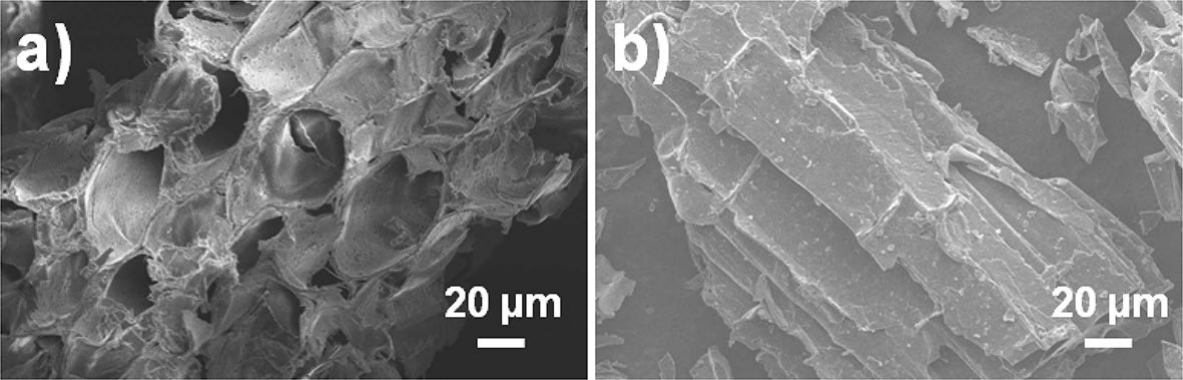
(**A,B**) Unloaded and phenol-loaded SBAC SEM micrographs.

The results of the scanning electron microscopy (SEM) study showed that the surface of the material under investigation exhibited a heterogeneous morphology, characterized by the presence of numerous pores. These pores tended to enhance the adsorption process, suggesting that they played a significant role in the material's ability to adsorb substances. This is supported by the observations made in Fig. 2A,B. Interestingly, after the adsorption of phenol, the pore structure associated with the high surface area appeared to disappear, as shown in Fig. 2B. Instead, a smoother and more even surface was observed. This suggests that the adsorption of phenol led to changes in the surface morphology of the material, potentially due to the filling or blocking of the pores by the adsorbed phenol molecules^39^. The introduction of ZnCl_2_ chemical activation caused the formation of new micropores and the expansion of existing micropores. This resulted in an increase in the volume and surface area of the micropores. During the carbonization process, the pores were generated as a result of the evaporation of ZnCl_2_. The activation with ZnCl_2_ led to the development of a significant number of micropores, which played a crucial role in the removal of pollutants from the solution^40^.

**Figure 3:**
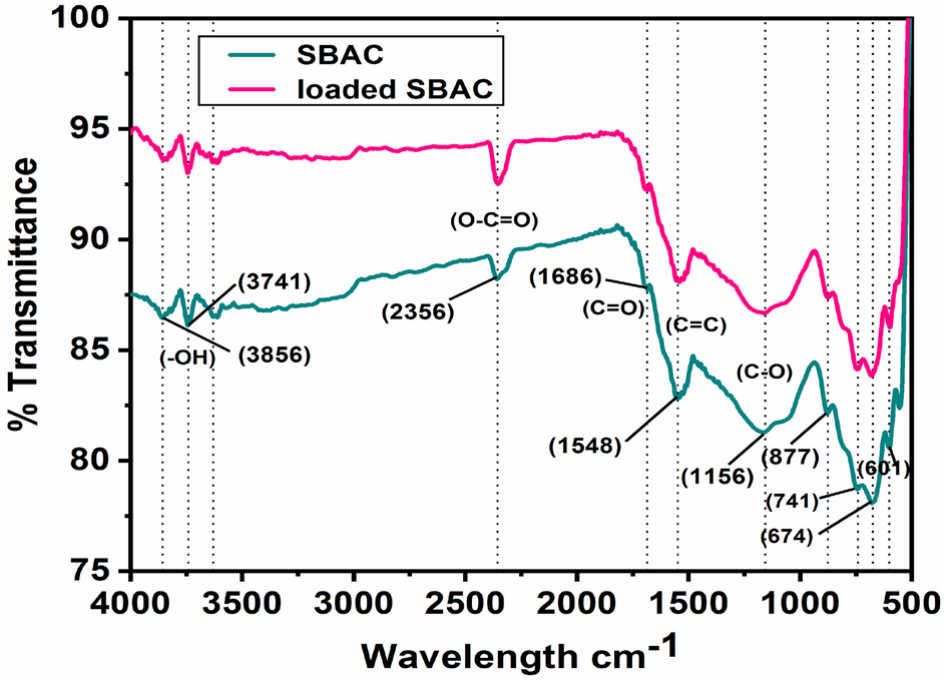
Unloaded and phenol-loaded SBAC FTIR spectra.

The FTIR spectrum before and after phenol adsorption on the surface of SBAC revealed certain changes in the spectral bands as shown in Fig. 3. Here is a breakdown of the observed changes and their corresponding functional groups: these dips in the spectrum at 3856 and 3714 cm^−1^ indicate the presence of –OH functional groups, specifically phenol, alcohol, and carboxylic acid groups^41^, dip at 2356 cm^−1^ corresponds to the O–C=O functional group^42^, dip at 1681 cm^−1^ attributed to the vibrations of C=O functional groups^43–45^, dip at 1542 cm^−1^ is associated with aromatic C=C bonds^46^, dip at 1175 cm^−1^ represents the ν C–O vibrations of phenols and ethers^47^, dips at 874 cm^−1^, 808 cm^−1^, 746 cm^−1^, 679 cm^−1^ are signifies the presence of isolated hydrogen, two adjacent hydrogens, four adjacent hydrogens, and five adjacent hydrogens respectively^48,49^. These observations in the FTIR spectrum before and after adsorption provide valuable information about the functional groups present in SBAC and the changes that occur as a result of the adsorption process.

**Figure 4:**
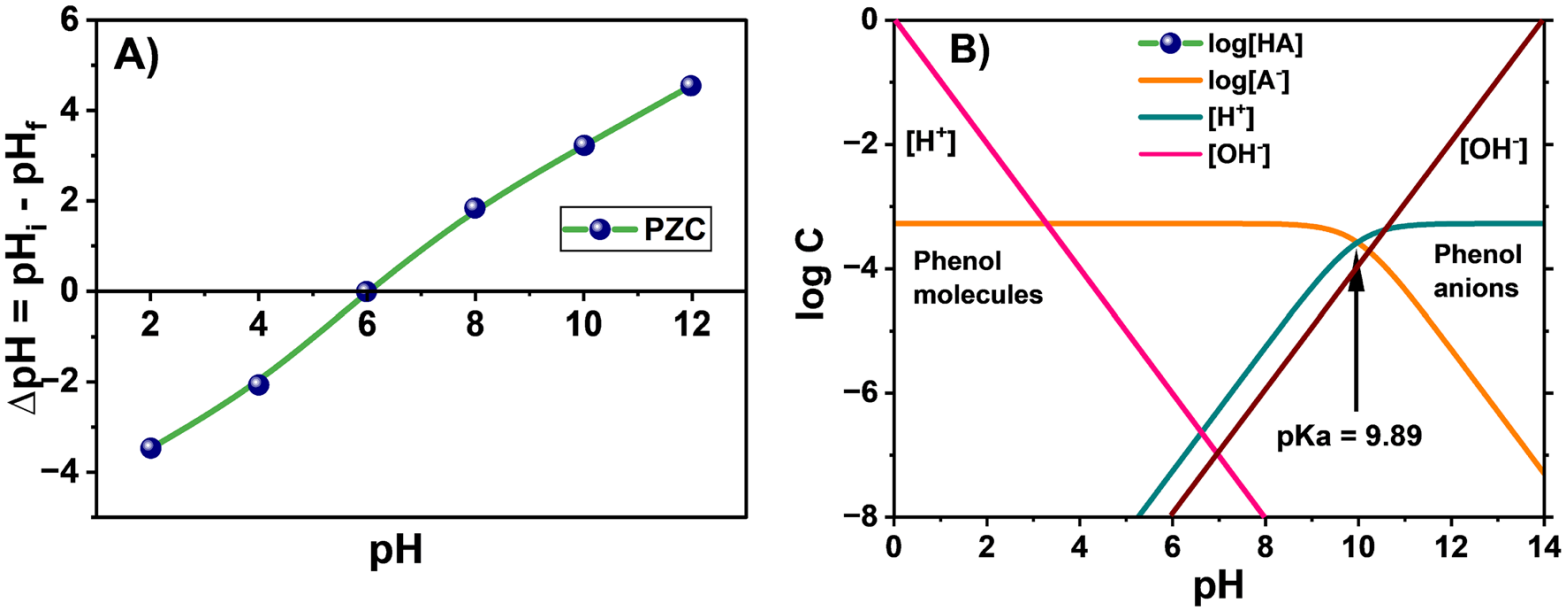
(**A**) PZC of SBAC and (**B**) Phenol log C-pH diagram.

The pH at which adsorbent surface posses zero charge know as point of zero charge (PZC). PZC of SBAC takes place at pH 6 as shown in Fig. 4A. Decreasing the pH < 6 causes positive charge and increasing the pH > 6 causes adsorbent surface negative on the adsorbent surface. Phenol was a very weak acid with the pK_a_ value of 9.89, which was dissociated if the pH value exceeds pK_a_ and at low pH it was mainly in molecular state as shown in log C-pH graph Fig. 4B. With higher pH values, the removal of phenol decreases because of phenol ionization and repugnance between phenolate anions and negative SBAC sites^50^. At lesser pH (< PZC), phenol molecules get easily attached onto the negative SBAC surface and moreover the pH of the phenol less than the PZC i.e., 5.5 < 6. As a result, at pH 5.5 the maximum removal of phenol takes place^4^.

### L_9_ Orthogonal array experimental results

Batch study is carried out for the removal of phenol onto SBAC at particular conditions which are listed in the Table S3. Three times each experiment is conducted and average q_t_ value is employed for the Taguchi’s analysis. In Table S5, the results of experimental runs, including S/N ratio values determined under larger the better conditions, are reported.

### Effect of adsorption parameters

**Figure 5:**
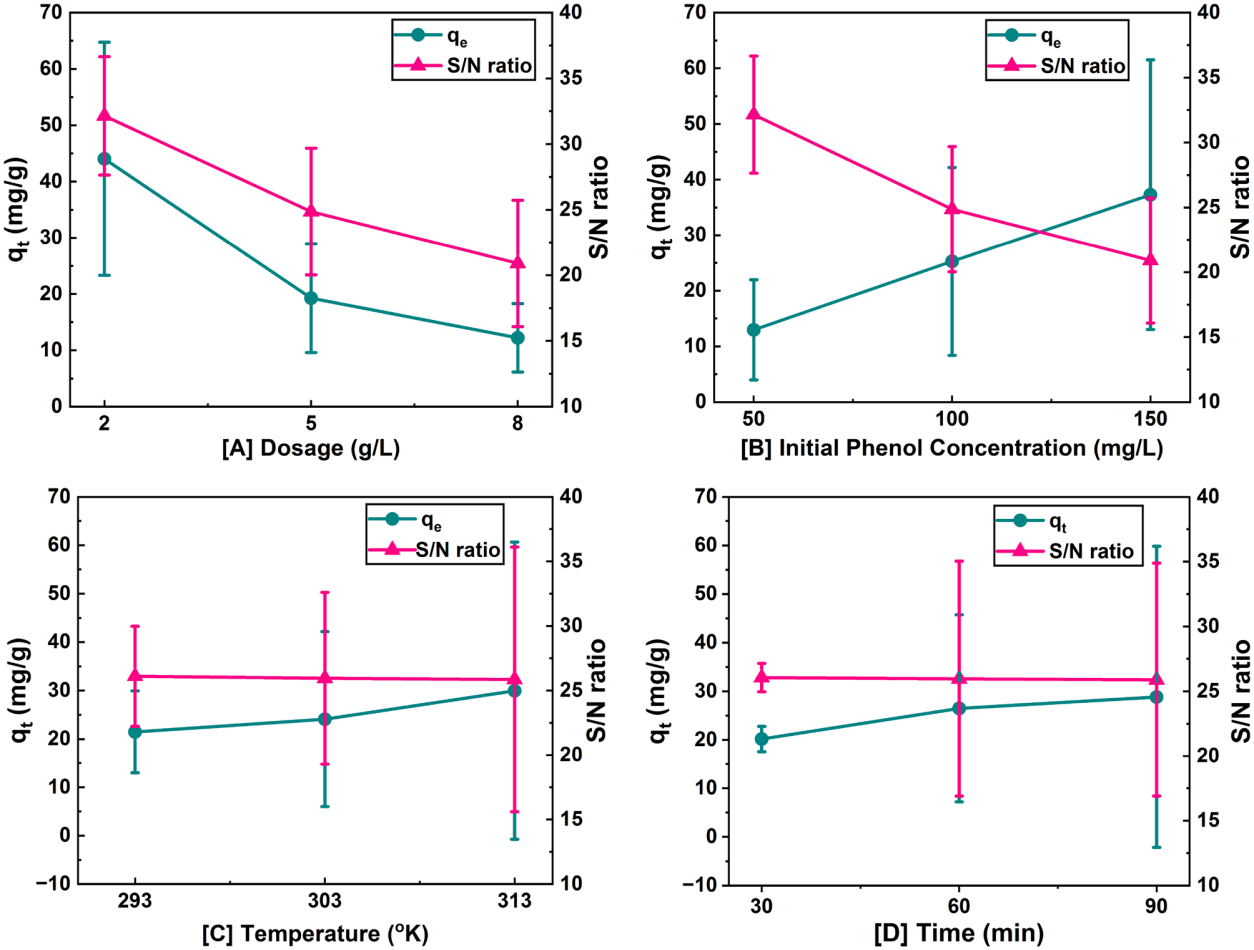
Effect of adsorption parameters on adsorption capacity of SBAC and S/N ratio for phenol removal onto SBAC.

Adsorption capacity is the primary response characteristic for optimization, and these values are controlled by process factors like m, C_0_, T, and t given in Table S5 at various levels. The fact that factor A had the greatest effect at level 1 means that altering the value of parameter A at level 1 had a considerable impact on the q_t_ values, as shown by Fig. 5 and Table S6. However, for the adsorbent SBAC, variables B, C, and D had the greatest influence at level 3. The influence of each level relative to the others was determined by comparing levels 1 and 2 (L_2_–L_1_) and levels 2 and 3 (L_3_–L_2_). A larger disparity between two levels signifies a higher influence of that level. In this case, Table S6 suggests that parameter B (C_0_) had the greatest influence on the q_t_ values. The q_t_ values increased as C_0_ increased, which can be attributed to a higher mass driving force and a lesser barrier to the absorption of phenol onto SBAC^37^).

Figure 5 shows that as the levels of parameter A (m) increase from level 1 to 3, denoted as m, the sorption capacity decreases from 44.04 to 12.21 mg/g. This decrease is attributed to a decrease in the phenol to SBAC ratio. In other words, as the level of parameter A increases, there is a larger amount of adsorbent available compared to the amount of phenol, resulting in a lower sorption capacity. As the levels of parameter B (C_0_) increase from level 1 to 3, the sorption capacity increases from 12.97 to 37.27 mg/g. This increase is attributed to an increase in the mass transfer driving force and a lesser barrier to the absorption of phenol onto SBAC. In simpler terms, higher levels of parameter B create conditions that facilitate the sorption process, such as enhancing the mass transfer of phenol molecules onto SBAC and reducing barriers to the adsorption process. As the levels of parameter C (T) increase from level 1 to 3, the sorption capacity increases from 21.47 to 29.95 mg/g. Higher temperatures result in lower phenol viscosity, increased molecule mobility, and higher kinetic energy. These conditions enhance the chances of phenol molecules adsorbing onto SBAC and increase the diffusion rate of phenol, leading to an increased sorption capacity. The sorption capacity of phenol onto SBAC changes as the parameter D (t) increases from level 1 to 3. The sorption capacity of SBAC first increases quickly from level 1 to 2, (20.19 to 26.49 mg/g). This increase is attributed to the abundance of large unoccupied sites on SBAC for the sorption of phenol. i.e., when the contact time is increased from level 1 to 2, more vacant sites become accessible for phenol molecules to adsorb onto SBAC, resulting in a higher sorption capacity. However, in the later phase, as the contact time increases further from level 2 to 3 (26.49 to 28.84 mg/g), the availability of vacant sites on SBAC decreases. This reduction in available vacant sites hampers the adsorption capacity of phenol, causing a retardation in the sorption process. Consequently, the increase in adsorption capacity from level 2 to 3 for SBAC is only 2.35 mg/g, which is relatively low compared to the previous increase observed.

**Figure 6:**
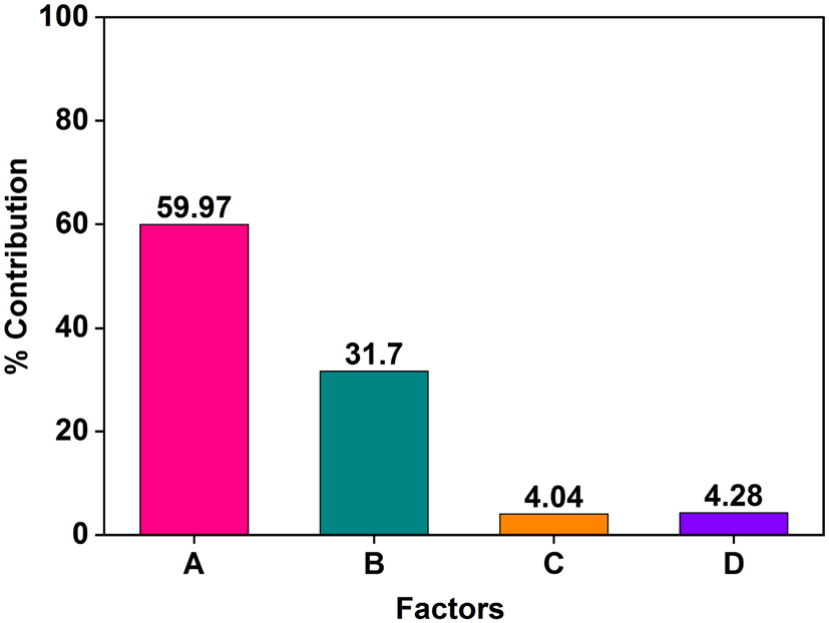
The percentage of each parameter's contribution to the adsorption capacity of SBAC for phenol.

The percentage contributions of a different parameters to the overall sorption of phenol over SBAC are shown in Fig. 6 and Table S7. The parameter A (m) has the highest contribution of 59.97% to the sorption process. It indicates that the amount or dosage of the adsorbent plays a significant role in the sorption of phenol as increasing the adsorbent dose can enhance the sorption efficiency. The second most influential parameter is B (C_0_) with a contribution of 31.70%. This suggests that the concentration of phenol in the initial solution affects its sorption onto SBAC. Higher initial concentrations may result in reduced sorption efficiency. The parameter D (t) has a relatively lower contribution of 4.28%. This indicates that the contact time between the phenol solution and SBAC influences the sorption process to a lesser extent compared to factors A and B as longer contact times may lead to increased sorption. The parameter C (T) with the least effect on the sorption process with a contribution of only 4.04%. This suggests that temperature plays a minor role in the sorption of phenol onto SBAC. However, it's important to note that even though its contribution is low, temperature can still impact the kinetics and thermodynamics of the sorption process.

### Selection of optimized level and estimation of optimized response characteristics

Optimization study was conducted to determine the optimal levels of parameters A, B, C, and D for maximizing the response value (q_t_) in a process. The study involved analyzing a response curve (Fig. 5) and Table S6 to identify the factor levels that produced the highest q_t_ values for SBAC. The results indicate that the first level of Parameter A (m) and the third level of parameters B (C_0_), C (T), and D (t) yielded maximum values of q_t_. The average values of q_t_ (mg/g) for these optimal levels are as $\overline{A}_{1}$ = 44.04, $\overline{B}_{3}$ = 37.27, $\overline{C}_{3}$ = 29.95 and $\overline{D}_{3}$ = 28.84. The grand total of all the qt results (T) is reported as 679.66 mg/g, and the total number of results (N) is given as 27. These values provide an overview of the performance of the optimization study and the distribution of q_t_ values obtained. Put the all these values in Eq. (6) becomes *µ*_*SBAC*_ is 64.59 mg/g. Keep values N is 27 and total DOF associated in the estimate of mean is 2 in Eq. (9), becomes *n*_*eff*_ is 3. To calculate the confidence interval of 95% for the population mean and the confirmation experiments, certain values are required such as N is 27, the degrees of freedom error *f*_*e*_ is 18, the recalculated error variance after pooling from Table S8 V_e_ is 16.97 mg/g and F_0.05_ (1,18) is 4.4139 (from standard F-distribution table). Hence Eqs. 7 and 8 are becomes CI_POP_ =  ± 4.99 and CI_CE_ =  ± 5.26, and all these values are presented in Table S8.

### Confirmation experiment

There is already an A_1_B_3_C_3_D_3_ run exists in OA and has been assigned as run number 3. Table S3 shows that the average sorption capacity of the SBAC material for the three repetitions is 64.59 mg/g. Also, this average sorption capacity value is within the range shown in Table S8 for the 95% confidence interval (CI). These results suggest that Taguchi's technique for optimizing process parameters can be used to enhance sorption.

### Adsorption isotherms

**Figure 7:**
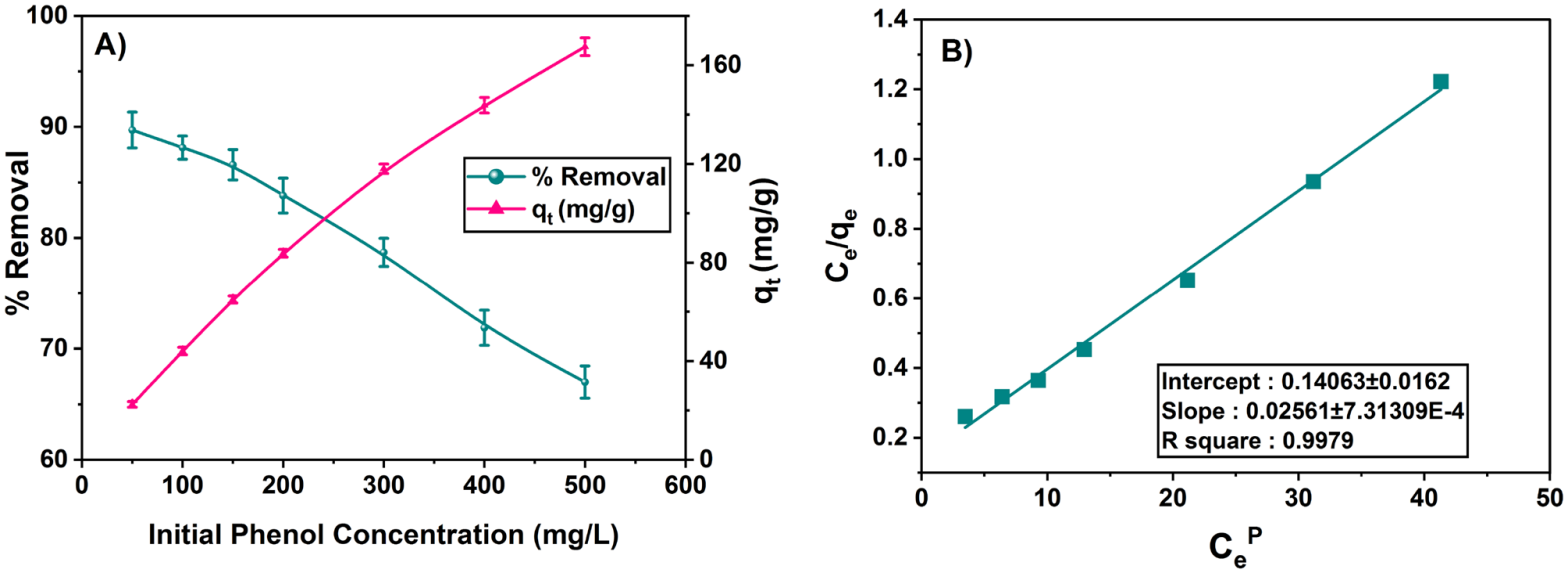
(**A**) Adsorption capacity of SBAC with varying initial phenol concentration during isotherm study and (**B**) Radke-Prausnitz isotherm plot for phenol adsorption onto SBAC (for initial phenol concentration: varying 50 to 500 mg/L, contact time: 90 min, pH: 5.5, temperature: 313 K, and speed: 150 rpm).

The adsorption isotherm describes the equilibrium interactions between the phenol molecules and the surface of SBAC. These studies provide insights into the sorption behavior and help in the design of sorption systems^51^. Different adsorption isotherms can be obtained by varying the initial phenol content and monitoring the ensuing adsorption at constant mixing conditions, including pH (5.5), temperature (313 K), speed (150 rpm), time (90 min), and amount of adsorbent (2 g/L). Figure 7A illustrates the SBAC adsorption capacity with the varying initial phenol concentration and the phenol removal rate in percentage.

Batch experimental results showed that adsorption capacity of SBAC increases from 22.48 to 166.95 mg/g as the initial phenol concentration increases from 50 to 500 mg/L. This increase in sorption capacity is likely due to the presence of more phenol ions at higher adsorbate concentrations^52^. To determine the best-fitted isotherm model for the sorption of phenol onto the adsorbent SBAC, several isotherm models were considered, including Freundlich^53^, Langmuir^54^, Temkin^55^, Toth^56^, Redlich-Peterson^57^ and Radke-Prausnitz^58^.

In addition to the correlation coefficient (R^2^), various error analysis functions were used to assess the suitability of the isotherm models^4^. These error functions include hybrid fractional error function (HYBRID), MPSD (Marquardt's Percent Standard Deviation), ARE (Average Relative Error), SAE (Sum of Absolute Errors), χ2 (Chi-square), and SSE (Sum of Squares of Errors). These error functions values are shown in the Table S9 and lower values of these error analysis functions indicate a better fit of the isotherm model^59^. The Radke-Prausnitz isotherm model exhibited lower values of error functions among other isotherm models (HYBRID = − 0.941, MPSD = 5.129, ARE = 4.047, SSE = 39.865, χ^2^ = 0.560, SAE = 15.295) and yielded a higher regression coefficient (R^2^ = 0.9979) compared to the other models, which shows that phenol removal onto SBAC has been thoroughly explained as shown in Fig. 7B.

### Adsorption kinetics

**Figure 8:**
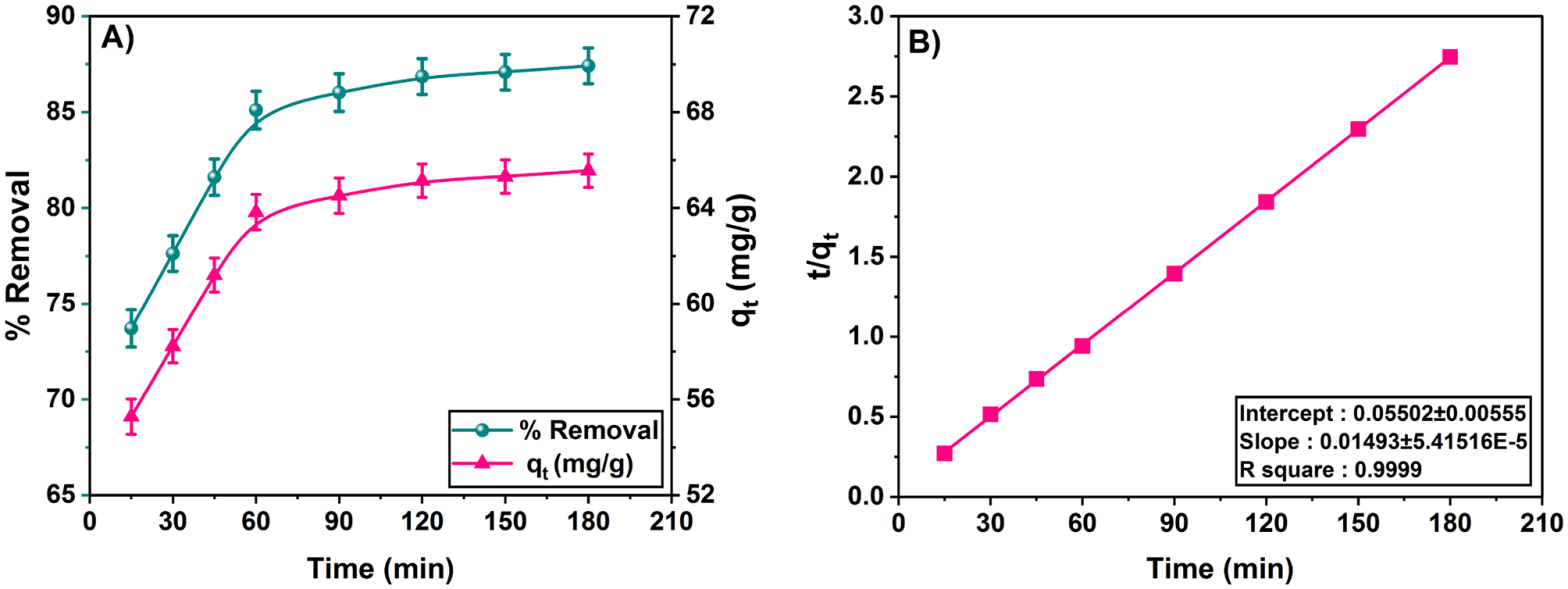
(**A**) Effect of contact time on phenol adsorption: C_0_ = 150 mg/L, m = 2 g/L, T = 313 K and 150 rpm, V = 50 mL, and pH = 5.5 and optimum contact time found to 90 min and (**B**) pseudo second-order kinetic model phenol removal: C_0_ = 150 mg/L, m = 2 g/L, t = 0–180 min, 150 rpm, V = 50 mL, and pH = 5.5 and T = 303 K.

The study of adsorption of kinetics gives the better understanding of adsorption mechanism of phenol onto the SBAC. The study performed by varying the contact time between phenol and adsorbent from 0 to 180 min at 313 K temperature, 2 g/L adsorbent dosage, 150 rpm, pH 5.5, and initial phenol concentration of 150 mg/L as shown in Fig. 8A. The results of experiment show that the amount of phenol removed (% Removal) and amount of phenol adsorbed onto SBAC both rise with contact time until equilibrium is reached. Initially adsorption takes place at high rate due to availability of abundance of active sites on the adsorbent. Thereafter, an increase in contact time has no effect on phenol adsorption because the number of active sites decreases as contact time increases^60^. The study carried out for 180 min, for an initial phenol concentration (C_0_) of 150 mg/L, approximately 64.5 mg/g and 86% of phenol was adsorbed within the first 90 min and after that there is marginal difference in removal of phenol. Therefore, the optimum contact time for the phenol adsorption on SBAC is 90 min. Some of the earlier studies reports that optimum time is 30 min for ZnO Nano catalyst^10^, 60 min for banana peels^4^, 120 min for guava tree bark^20^, and 120 min wood charcoal^61^.

In the present study, pseudo-first-order and pseudo-second-order kinetic models^62^ are used to analyze the kinetic data and presented in Table S10. When these models are compared, the regression value obtained for the pseudo-second-order model (R^2^ = 0.9999) is comparatively higher than that first order which suggests that pseudo–second-order kinetic model suitable for the adsorption phenol onto SBAC (Fig. 8B). Some earlier studies also cited the pseudo-second-order model as the best fit model for the phenol removal on chitin^63^, babul saw dust^23^, and scoria stone^16^.

### Adsorption thermodynamics

Thermodynamic analysis is carried out for the phenol adsorption by increasing the temperature from 283 to 323 K while maintaining the same contact time of 90 min, adsorbent dosage of 2 g/L, and initial phenol concentration of 150 mg/L, phenol pH of 5.5 and agitation speed of 150 rpm. Thermodynamics parameters can be calculated out by using distribution coefficient, *K*_*ads*_ which is dependent on temperature. In our study, the Radke–Prausnitz is found to be the best fit isotherm. Hence, the values of constant K_RP_ viz. 0.095, 0.258, 0.182, 0.148 and 0.174 L/g at respective temperatures of 283, 293, 303, 313 and 323 K are calculated from the Radke-Prausnitz plot as shown in the Table S11.

The Gibbs free energy change equation^64^

10 $$\Delta G_{ads}^{0} = - RT\ln K_{ads}$$

11 $$\Delta G_{ads}^{0} = \Delta {\text{H}}^{0} - {\text{T}}\Delta {\text{S}}^{0}$$

**Figure 9:**
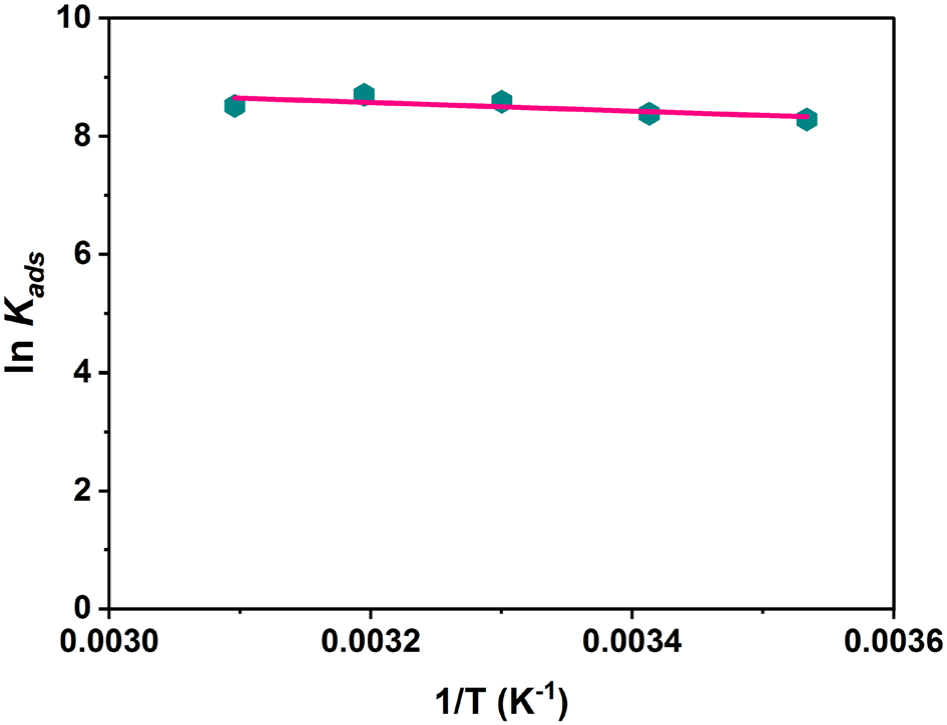
Thermodynamics Van’t Hoff plot ln *K*_*ads*_ vs. 1/T of SBAC for Radke-Prausnitz isotherm.

Equating the above two equations, we get

12 $$\ln K_{ads} = \frac{{\Delta G_{ads}^{0} }}{RT} = \frac{{\Delta {\text{S}}^{0} }}{R} - \frac{{\Delta {\text{H}}^{0} }}{RT}$$

where, *ΔG*^*0*^_*ads*_ Gibbs free energy change (kJ/mol), *T* Temperature (K), *ΔS*^*0*^ change of entropy (J/mol K), *R* Universal gas constant, *ΔH*^*0*^ change of enthalpy (kJ/mol), and K_ads_ distribution coefficient (L/mol). A plot of ln *K*_*ads*_ against 1/T, as illustrated in Fig. 9, gave a linear relationship with *ΔH*^*0*^ and *ΔS*^*0*^ evaluated from the slope and intercept, respectively, of the Van’t Hoff plot and *∆G*^*0*^_*ads*_ was calculated using Eq. (12). The thermodynamic parameters for the adsorption of phenol onto SBAC are shown in Table S11.

The values of *ΔG*^*0*^_*ad*s_ fall within the range of − 19.599 to − 23.222 kJ/mol. These values suggest that the sorption process approaches physisorption (physical adsorption) followed by chemisorption (chemical adsorption). Physisorption is indicated by *ΔG*^*0*^_*ads*_ values in the range of 0 to − 20 kJ/mol, while chemisorption is indicated by values in the range of − 80 to − 400 kJ/mol. It is observed that the values of *ΔG*^*0*^_*ads*_ become more negative (higher in magnitude) with an increase in temperature. This indicates that the driving force for the sorption process increases at higher temperatures. The negative values of *ΔG*^*0*^_*ads*_ obtained in the analysis indicate that the sorption process is thermodynamically favorable for the adsorption of phenol onto SBAC^65^. The values of *ΔH*^*0*^, the standard enthalpy change, were determined to be negative (− 6.035 kJ/mol). This suggests that the sorption process is exothermic. The values of *ΔS*^*0*^, the standard entropy change, were found to be positive (0.09 kJ/mol K). This indicates that the adsorption process leads to an increase in the stability of the adsorption phase. Positive *ΔS*^*0*^ values imply greater randomness or disorder at the molecular level, contributing to the overall stability of the adsorbed species.

### Adsorption mechanism

**Figure 10:**
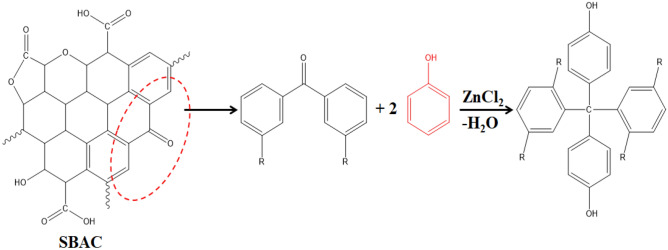
Plausible chemical interaction towards phenol adsorption.

The feasible mechanism is based on a thorough understanding of all conceivable physical and chemical interactions between the substrate (phenol) and adsorbent (SBAC), as evidenced by characterization data and experimental results. Due to van der Waals forces, chemical affinity, electrostatic attraction and a number of different functional groups, such as hydroxyl, carbonyl, lactone, ketone, aldehyde, etc., are present on the surface of activated carbon and contribute to the formation of physical and chemical bonds with phenol during the adsorption process^66^. The emulous behavior of ZnCl_2_-treated activated carbon could be attributed to additional chemical interactions via Lewis acid catalyzing reactions (Fig. 10) between nucleophilic groups and aromatic hydrocarbon, ketone or aldehyde groups^67^.

## Conclusions

The present study focuses on using Taguchi's design of experiment approach to optimize the processes parameters for the removal of phenol onto SBAC from an aqueous solution. Batch experimental results shows that the highest adsorption capacity of SBAC achieved around 64.59 mg/g under the optimum conditions is found (m = 2 g/L, C_0_ = 150 mg/L, t = 90 min and T = 313 K). The ANOVA study shows the adsorbent dosage has maximum influence (59.97%) and temperature has minimal influence (4.04%) on adsorption capacity of SBAC for the removal of phenol. For SBAC, the optimized parameters at various levels were determined to be A_1_, B_3_, C_3_, and D_3_ and the q_t_ value given by the Taguchi's approach and the experiment is almost same i.e., 64.59 mg/g. Therefore, the confirmation experiment (run number 3) shows that the adsorption capacity of SBAC values within the range of 95% CI_CE_. An isotherm study shows that Radke-Prausnitz isotherm model is well fitted to the equilibrium data obtained from experimental programme. Kinetic study raveled that pseudo-second-order kinetic model exhibited the best fit to the experimental data. Thermodynamic study (*ΔH*^*0*^ = − 6.035 kJ/mol and *ΔS*^*0*^ = 0.090 kJ/mol K) shows that the nature of adsorption process is random, spontaneous, exothermic and (*ΔG*^*0*^_*ad*s_ = − 19.599 to − 23.222 kJ/mol) suggesting that the adsorption process approaches physisorption followed by chemisorption. The BET surface area of SBAC before and after phenol adsorption are 415.96 and 78.33 m^2^/g, and observed surface area decreased by 81.17% after adsorption indicating the better sorption capacity SBAC. SEM study showed that the surface of adsorbent exhibited a heterogeneous morphology, characterized by the presence of numerous pores improves the adsorption, and proximate analysis of adsorbent determined consists 65.33% of fixed carbon improves the potential of the adsorbent. FTIR study shows that presence of hydroxyl, carbonyl, lactones, ketone, and aldehyde groups are responsible for phenol removal. Hence the application of Taguchi orthogonal array design is a cost-effective and time-efficient approach for carrying out experiments and optimizing procedures for adsorption of phenol by SBAC.

### Supplementary Information

Supplementary Information.

## Data Availability

The datasets generated during and/or analyzed during the current study are available from the corresponding author on reasonable request.
