# Supplementary material for: Adsorption of phenol using adsorbent derived from Saccharum officinarum biomass: optimization, isotherms, kinetics, and thermodynamic study
Source: Sci Rep. 2023 Oct 26;13:18356. doi: 10.1038/s41598-023-42461-y (PMC10603077; doi:10.1038/s41598-023-42461-y)
Supplement: Supplementary file 1 — Supplementary Information. [file 41598_2023_42461_MOESM1_ESM.docx]

# Supporting Information

# Adsorption of phenol using adsorbent derived from *saccharum officinarum* biomass: Optimization, isotherms, kinetics, and thermodynamic study

Upendra R. Darla^a^, Dilip H. Lataye^a *^, Anuj Kumar^b^, Bidhan Pandit^c^, Mohd Ubaidullah^d^

^a^ Department of Civil Engineering, Visvesvaraya National Institute of Technology, Nagpur, India 440010

^b^ Department of Chemistry, GLA University, Mathura-281406, India

^c^ Department of Materials Science and Engineering and Chemical Engineering, Universidad Carlos III de Madrid, Avenida de la Universidad 30, 28911 Leganés, Madrid, Spain

^d^ Department of Chemistry, College of Science, King Saud University, P.O. Box 2455, Riyadh 11451, Saudi Arabia

**Table S1** Chemical and its details

| **Chemical name** | **Molecular formula** | **CAS number** | **Company** | **Assay** |
| --- | --- | --- | --- | --- |
| Phenol | C_6_H_5_OH | 108-95-2 | Qualigens | 99.5% |
| Zinc chloride solution | ZnCl_2_ | 7646-85-7 | Loba Cheme | 0.1M |
| Sodium hydroxide | NaOH | 1310-73-2 | Merck | ≥97% |
| Hydrochloric acid | HCl | 7647-01-0 | Qualigens | 35.8-38% |

**Table S2** Adsorption parameters and its levels

| **Parameters** | | | **Units** | **Levels (L)** | | |
| --- | --- | --- | --- | --- | --- | --- |
|  |  |  |  | **1** | **2** | **3** |
| A | Adsorbent dose | m | g/L | 2 | 5 | 8 |
| B | Initial phenol concentration | C_0_ | mg/L | 50 | 100 | 150 |
| C | Temperature | T | °C | 20 | 30 | 40 |
| D | mixing time | t | min | 30 | 60 | 90 |

**Table S3** Taguchi’s L_9_ OA and DOE for phenol adsorption onto SBAC

| **Run** | **Taguchi Orthogonal Arrays for L_9_ (3^4^)** | | | |  | **Design of experiments Sets for SBAC** | | | |
| --- | --- | --- | --- | --- | --- | --- | --- | --- | --- |
|  | **Combinations** | | | |  | **Parameters** | | | |
|  | **A** | **B** | **C** | **D** |  | **A** | **B** | **C** | **D** |
|  | **m** | **C_0_** | **T** | **t** |  | **m** | **C_0_** | **T** | **t** |
| 1 | 1 | 1 | 1 | 1 |  | 2 | 50 | 20 | 30 |
| 2 | 1 | 2 | 2 | 2 |  | 2 | 100 | 30 | 60 |
| 3 | 1 | 3 | 3 | 3 |  | 2 | 150 | 40 | 90 |
| 4 | 2 | 1 | 2 | 3 |  | 5 | 50 | 30 | 90 |
| 5 | 2 | 2 | 3 | 1 |  | 5 | 100 | 40 | 30 |
| 6 | 2 | 3 | 1 | 2 |  | 5 | 150 | 20 | 60 |
| 7 | 3 | 1 | 3 | 2 |  | 8 | 50 | 40 | 60 |
| 8 | 3 | 2 | 1 | 3 |  | 8 | 100 | 20 | 90 |
| 9 | 3 | 3 | 2 | 1 |  | 8 | 150 | 30 | 30 |

**Table S4.** SBAC proximate analysis

| **Sr. No.** | **Characteristics** | **SBAC (%)** |
| --- | --- | --- |
| 1 | Moisture content | 5.65 |
| 2 | Ash content | 8.13 |
| 3 | Volatile matter | 20.88 |
| 4 | Fixed carbon | 65.33 |

**Table S5**. Taguchi L_9_ Orthogonal array with experimental q_t_ and S/N ratio values

| **Adsorbent** | **Run** | **Factors** | | | |  | **Experimental results** | | | **S/N Ratio** |
| --- | --- | --- | --- | --- | --- | --- | --- | --- | --- | --- |
|  |  | **A** | **B** | **C** | **D** |  | **q_t_ (mg/g)** | | |  |
|  |  | **m** | **C_0_** | **T** | **t** |  | **R_1_** | **R_2_** | **R_3_** |  |
| SBAC | 1 | 1 | 1 | 1 | 1 |  | 23.15 | 23.16 | 23.15 | 27.29 |
|  | 2 | 1 | 2 | 2 | 2 |  | 44.39 | 44.36 | 44.38 | 32.94 |
|  | 3 | 1 | 3 | 3 | 3 |  | 64.59 | 64.60 | 64.57 | 36.20 |
|  | 4 | 2 | 1 | 2 | 3 |  | 9.64 | 9.65 | 9.65 | 19.69 |
|  | 5 | 2 | 2 | 3 | 1 |  | 19.18 | 19.18 | 19.17 | 25.65 |
|  | 6 | 2 | 3 | 1 | 2 |  | 28.97 | 28.98 | 28.99 | 29.24 |
|  | 7 | 3 | 1 | 3 | 2 |  | 6.10 | 6.10 | 6.09 | 15.71 |
|  | 8 | 3 | 2 | 1 | 3 |  | 12.29 | 12.28 | 12.28 | 21.79 |
|  | 9 | 3 | 3 | 2 | 1 |  | 18.28 | 18.25 | 18.23 | 25.23 |

Where R_1_, R_2_, R_3_ are the adsorption capacity of L_1_, L_2_ and L_3_ for each run

**Table S6** Average and main effects of q_t_ values for SBAC: Raw and S/N data Adsorbent

| **Adsorbent** | **Factors** | **Raw data,**  **average value** | | | **Main effect (raw data)** | | **S/N data, average value** | | | **Main effect (S/N data)** | |
| --- | --- | --- | --- | --- | --- | --- | --- | --- | --- | --- | --- |
|  |  | **L_1_** | **L_2_** | **L_3_** | **L_2_-L_1_** | **L_3_-L_2_** | **L_1_** | **L_2_** | **L_3_** | **L_2_-L_1_** | **L_3_-L_2_** |
| SBAC | A | **44.04** | 19.27 | 12.21 | - 24.77 | -7.06 | 32.15 | 24.86 | 20.91 | -7.28 | -3.95 |
|  | B | 12.97 | 25.28 | **37.27** | 12.31 | 11.99 | 20.90 | 26.79 | 30.22 | 5.90 | 3.43 |
|  | C | 21.47 | 24.09 | **29.95** | 2.62 | 5.86 | 26.11 | 25.95 | 25.85 | -0.15 | -0.10 |
|  | D | 20.19 | 26.49 | **28.84** | 6.29 | 2.35 | 26.06 | 25.96 | 25.89 | -0.09 | -0.07 |

**Table S7** ANOVA for phenol adsorption on SBAC

| **Adsorbent** | **Factors** | **ANOVA qt** | | | | | **Pooled ANOVA qt** | | | | |
| --- | --- | --- | --- | --- | --- | --- | --- | --- | --- | --- | --- |
|  |  | **S** | **DOF** | **V** | **F** | **P** | **S** | **DOF** | **V** | **F** | **P** |
| SBAC | A | 5029.27 | 2 | 2514.6 | 14470030.1 | 59.97 | 5029.3 | 2 | 2514.64 | 148.15 | 59.56 |
|  | B | 2658.64 | 2 | 1329.3 | 7649329.8 | 31.70 | 2658.6 | 2 | 1329.32 | 78.32 | 31.29 |
|  | C | 339.48 | 2 | **169.7** | 976725.2 | 4.05 | **0** | **0** | **0** | **0** | **0** |
|  | D | 359.55 | 2 | 179.8 | 1034484.1 | 4.29 | 359.55 | 2 | 179.78 | 10.59 | 3.88 |
|  | Error | 0.0031 | 18 | 0.0 | 1.00 | 0.00 | 339.48 | 20 | **16.97** | 1.00 | 5.26 |
|  | Totals | 8386.94 | 26 | **4193.5** | 14470030.1 | 100.00 | 8386.9 | 26 | **4040.71** |  | 100.00 |

S sum of squares, V variance, F variance ratio, and P % contribution

**Table S8** Predicted optimum q_t_ values, confidence intervals, and the confirmation experiment

| **Adsorbent** | **Optimum Level Process Parameters** | **Predicted Optimum Value (mg/g)** | **Confidence interval 95%** | **Average of Confirmation (mg/g)** |
| --- | --- | --- | --- | --- |
| SBAC | A_1_B_3_C_3_D_3_ | 64.59 | CI_POP_: 59.39 < µ_SBAC_ < 69.58 | 64.58 |
|  |  |  | CI_CE_: 59.32 < µ_SBAC_ < 69.85 |  |

**Table S9** Isotherm parameters and error analysis values of phenol onto SBAC (m = 2 g/L, 150 rpm, t = 90 min, pH = 5.5, and C_0_ = 50-500 mg/L)

| **Isotherms** | **Constants** | **Temperature (K)** | | | | |
| --- | --- | --- | --- | --- | --- | --- |
|  |  | **283** | **293** | **303** | **313** | **323** |
| **Langmuir** $\frac{C_{e}}{q_{e}}=\frac{C_{e}}{q_{m}}+\frac{1}{K_{L}q_{m}}$  | *q_m_ (mg/g)* | 136.986 | 172.414 | 192.308 | 208.333 | 172.414 |
|  | *K_L_ (L/g)* | 0.018 | 0.019 | 0.021 | 0.022 | 0.021 |
|  | *R^2^ (linear)* | 0.9937 | 0.9982 | 0.998 | 0.9964 | 0.9979 |
|  | *R^2^(non-linear)* | 0.997 | 0.999 | 0.999 | 0.998 | 0.999 |
|  | *HYBRID* | 1.267 | 0.677 | 0.200 | 0.5498 | 0.150 |
|  | *MPSD* | 7.527 | 3.546 | 3.725 | 4.3594 | 3.570 |
|  | *SSE* | 63.531 | 25.051 | 35.848 | 59.3532 | 46.378 |
|  | *SAE* | 18.157 | 11.432 | 13.540 | 17.503 | 12.482 |
|  | *ARE* | 5.354 | 2.690 | 3.006 | 3.6017 | 2.685 |
|  | *χ2* | 1.094 | 0.300 | 0.388 | 0.5723 | 0.483 |
| **Freundlich** $q_{e}=K_{F}C_{e}^{1/n}$  | *K_F_ (L/g)* | 8.729 | 8.835 | 10.006 | 10.650 | 9.757 |
|  | *n* | 2.079 | 1.859 | 1.825 | 1.780 | 1.908 |
|  | *1/n* | 0.481 | 0.538 | 0.548 | 0.562 | 0.524 |
|  | *R^2^ (linear)* | 0.9746 | 0.9675 | 0.9712 | 0.9748 | 0.9608 |
|  | *R^2^(non-linear)* | 0.987 | 0.984 | 0.985 | 0.987 | 0.980 |
|  | *HYBRID* | -0.547 | -0.920 | -0.831 | -0.761 | -1.0772 |
|  | *MPSD* | 10.648 | 15.248 | 13.190 | 12.606 | 14.9683 |
|  | *SSE* | 275.660 | 593.855 | 732.700 | 735.207 | 687.2270 |
|  | *SAE* | 37.734 | 53.179 | 57.521 | 58.911 | 55.348 |
|  | *ARE* | 9.444 | 12.024 | 11.558 | 11.214 | 12.7094 |
|  | *χ2* | 3.471 | 5.820 | 6.355 | 6.094 | 6.9181 |
| **Temkin** $q_{e}=B\ln C_{e}+B\ln K_{T}$  | *B* | 27.784 | 35.78 | 39.16 | 42.12 | 35.41 |
|  | *K_T_ (L/g)* | 0.219 | 0.213 | 0.246 | 0.266 | 0.239 |
|  | *R^2^ (linear)* | 0.9896 | 0.9928 | 0.9889 | 0.9847 | 0.9948 |
|  | *R^2^(non-linear)* | 0.995 | 0.996 | 0.994 | 0.992 | 0.997 |
|  | *HYBRID* | 1.961 | 2.719 | 3.603 | 4.486 | 2.239 |
|  | *MPSD* | 12.107 | 14.728 | 18.178 | 21.754 | 10.840 |
|  | *SSE* | 75.637 | 83.870 | 160.040 | 258.275 | 61.809 |
|  | *SAE* | 19.282 | 21.040 | 28.870 | 35.900 | 18.732 |
|  | *ARE* | 7.743 | 8.060 | 11.009 | 13.073 | 6.767 |
|  | *χ2* | 2.367 | 2.894 | 6.331 | 10.419 | 1.911 |
| **Toth** $q_{e}=\frac{q_{Th}C_{e}}{[(1/K_{Th})+\mathrm{C}_{e}^{Th}]^{1/Th}}$  | *Th* | 0.461 | 0.261 | 0.577 | 0.413 | 0.412 |
|  | *q_Th_(mg/g)* | 246.304 | 1172.874 | 302.905 | 535.119 | 399.416 |
|  | *K_Th_ (mg/L)^Th^* | 0.185 | 0.345 | 0.109 | 0.200 | 0.214 |
|  | *R^2^ (linear)* | 0.9981 | 0.9833 | 0.9982 | 0.994 | 0.9909 |
|  | *R^2^(non-linear)* | 0.999 | 0.992 | 0.999 | 0.997 | 0.995 |
|  | *HYBRID* | -0.175 | -0.485 | -2.161 | -0.241 | -0.6200 |
|  | *MPSD* | 3.423 | 12.300 | 4.035 | 5.806 | 8.4904 |
|  | *SSE* | 25.271 | 164.992 | 41.628 | 72.620 | 90.0271 |
|  | *SAE* | 9.808 | 29.925 | 14.055 | 20.853 | 19.890 |
|  | *ARE* | 2.663 | 7.702 | 3.269 | 4.788 | 5.9344 |
|  | *χ2* | 0.356 | 2.094 | 0.417 | 0.887 | 1.4087 |
| **Redlich Peterson** $q_{e}=\frac{K_{R}C_{e}}{1+a_{R}C_{e}^{\text{β}}}$  | *K_R_ (L/g)* | 2.300 | 6.737 | 6.900 | 6.000 | 5.200 |
|  | *β* | 0.5189 | 0.6088 | 0.222 | 0.775 | 0.7284 |
|  | *a_R_(L/g)* | 0.264 | 0.319 | 0.222 | 0.091 | 0.126 |
|  | *R^2^ (linear)* | 0.9781 | 0.9762 | 0.9858 | 0.9972 | 0.9834 |
|  | *R^2^(non-linear)* | 0.989 | 0.988 | 0.993 | 0.999 | 0.992 |
|  | *HYBRID* | 45.517 | -0.299 | -0.132 | -0.153 | -0.2276 |
|  | *MPSD* | 42.183 | 11.370 | 6.807 | 2.962 | 6.8390 |
|  | *SSE* | 2668.748 | 312.984 | 269.392 | 59.140 | 191.6096 |
|  | *SAE* | 129.674 | 37.792 | 33.855 | 16.156 | 28.956 |
|  | *ARE* | 37.930 | 8.072 | 6.111 | 2.601 | 6.1518 |
|  | *χ2* | 72.858 | 2.878 | 2.165 | 0.463 | 1.8193 |
| **Radke Prausnitz** $q_{e}=\frac{K_{RP}k_{rp}C_{e}}{1+K_{RP}C_{e}^{P}}$  | *P* | 0.792 | 0.672 | 0.710 | 0.729 | 0.727 |
|  | *k_rp_(mg/g)/(mg/L)^1/P^* | 41.667 | 27.248 | 39.063 | 49.020 | 37.313 |
|  | *K_RP_ (L/g)* | 0.095 | 0.258 | 0.182 | 0.148 | 0.174 |
|  | *R^2^ (linear)* | 0.9986 | 0.9935 | 0.9959 | 0.9976 | 0.9955 |
|  | *R^2^(non-linear)* | 0.999 | 0.997 | 0.998 | 0.999 | 0.998 |
|  | *HYBRID* | -2.049 | -1.942 | -1.491 | -0.941 | -1.869 |
|  | *MPSD* | 3.081 | 12.912 | 7.188 | 5.129 | 9.300 |
|  | *SSE* | 27.652 | 82.216 | 62.321 | 39.865 | 67.731 |
|  | *SAE* | 11.068 | 20.770 | 18.919 | 15.295 | 18.262 |
|  | *ARE* | 2.700 | 6.472 | 5.387 | 4.047 | 5.796 |
|  | *χ2* | 0.326 | 1.455 | 0.950 | 0.560 | 1.268 |

**Table S10.** Kinetic parameters for phenol adsorption onto SBAC: C_0_ =150 mg/L, speed = 150 rpm, m = 2 g/L, T = 313Kand t = 0 - 180 min

| **pseudo-first-order** | | | | **pseudo-second-order** | | | |
| --- | --- | --- | --- | --- | --- | --- | --- |
| q_e,exp_  (mg/g) | q_e,cal_ (mg/g) | k_f_  (min^-1^) | R^2^ | q_e,exp_ (mg/g) | q_e,cal_  (mg/g) | k_s_  (g/mg min) | R^2^ |
| 64.53 | 24.06 | 0.038 | 0.9092 | 64.53 | 67.11 | 0.004 | 1.000 |

**Table S11.** Thermodynamic parameters for the phenol adsorption on SBAC

| ***ΔH^0^*(kJ/mol)** | ***ΔS^0^*(kJ/mol K)** | ***ΔG^0^_ads_*(kJ/mol)** | | | | |
| --- | --- | --- | --- | --- | --- | --- |
|  |  | **283K** | **293K** | **303K** | **313K** | **323K** |
| -6.035 | 0.090 | -19.599 | -20.505 | -21.411 | -22.317 | -23.222 |
